# Investigating toxicity and Bias in stable diffusion text-to-image models

**DOI:** 10.1038/s41598-025-12032-4

**Published:** 2025-08-26

**Authors:** Matthias Schneider, Thilo Hagendorff

**Affiliations:** 1 Hasso-Plattner Institute, University of Potsdam , Potsdam, Germany; 2 Interchange Forum for Reflecting on Intelligent Systems, University of Stuttgart, Stuttgart, Germany

**Keywords:** Stable diffusion, Image generation, AI safety, Toxicity, Bias, Harmful content, Generative AI, Mathematics and computing, Computer science

## Abstract

Text-to-image models are increasingly popular and impactful, yet concerns regarding their safety and fairness remain. This study investigates the ability of ten popular Stable Diffusion models to generate harmful images, including sexual, violent, and personally sensitive material. We demonstrate that these models respond to harmful prompts by generating inappropriate content, which frequently displays troubling biases, such as the disproportionate portrayal of Black individuals in violent contexts. Our findings demonstrate a complete lack of any refusal behavior or safety measures in the models observed. We emphasize the importance of addressing this issue as image generation technologies continue to become more accessible and incorporated into everyday applications.

**Supplementary Information:**

The online version contains supplementary material available at 10.1038/s41598-025-12032-4.

## Introduction

Generative artificial intelligence (AI) systems, meaning models that produce text, images, or other data modalities, are currently gaining significant traction whereby the use cases for those models grow steadily^1^. With every new model generation, their capabilities are improving while their respective outputs are increasingly becoming part of the infosphere^2^. Due to their societal as well as technological relevance, researchers refer to such models as foundation models^3^, with GPT or Stable Diffusion models being prime examples^4,5^. As their usage expands, safety considerations regarding those models become increasingly important^6^. Particularly, the generation of toxic, discriminatory, violent, pornographic, or otherwise harmful content should be avoided.

### Background and motivation

Many closed source foundation models like ChatGPT implement built-in safety filters and leverage additional content filtering tools that in most cases do not allow users to generate harmful content^4,7,8^. However, next to that, a large community is publishing open source models with generative capabilities that often lack any safety fine-tuning, despite ongoing discussions about the dual-use risks they present^9^. Among the most popular distribution platforms for such models are Hugging Face and Civit AI. Here, such models can be downloaded and run on private computers in a non-controllable environment, with no safety constraints limiting the generated content. This can span violent, pornographic, taboo, misleading, or otherwise fraudulent content. For minors, such content can have long-term impacts^10^, and also affect many fields of society in various negative ways by “polluting” public discourses and the infosphere in general^11^.

Despite the remarkable creative potential and widespread adoption of text-to-image models, their openness and ease of access also introduce significant societal risks. Open source platforms provide little to no oversight regarding who uses these models or for what purposes. This lack of guardrails means that anyone, including minors or individuals with malicious intent, can generate highly realistic, explicit, or violent imagery with minimal effort. The democratization of such powerful technology thus creates a double-edged sword: it empowers artistic and educational innovation, but simultaneously amplifies the risk of harm and the perpetuation of social biases. As these models increasingly shape online content and influence cultural narratives, there is an urgent need to systematically evaluate and address their capacity to generate toxic and biased material. Our study aims to provide empirical evidence of these risks, underscoring the importance of developing and implementing effective safety mechanisms within the generative AI ecosystem.

### Related research

The current research landscape on AI-based image generators stresses challenges surrounding safety, fairness, and privacy. Researchers have pointed out how these models can be used to produce harmful content^12^ and have demonstrated strategies for bypassing safety filters^13^. Others have proposed new frameworks to quantify safety and fairness in image generation models^14^. Moreover, numerous studies investigated biases in these models, for instance by analyzing how they represent gender and ethnicity when being prompted with different social and professional contexts^15,16^. Here, papers demonstrate that models often under-represent marginalized identities^17–19^. Furthermore, researchers show that image training datasets used to train text-to-image models often contain racist, sexist, or otherwise offensive material^20,21^.

### Proposal

Our paper aligns with previous research on safety issues in image generation models, examining the capacity of these models to produce various types of harmful content. We systematically investigate the safety and bias characteristics of ten widely used Stable Diffusion text-to-image models. Our methodology involves selecting the most downloaded models from two leading platforms—Hugging Face and Civit AI—and subjecting them to a battery of prompts designed to elicit potentially harmful outputs, including sexual, violent, and personally sensitive content. We then analyze the generated images both manually and through automated classifiers to quantify the prevalence of harmful content and identify patterns of bias, particularly regarding race and gender. Through this empirical approach, we aim to assess not only the degree to which these models are capable of generating unsafe images but also to uncover embedded biases, content-wise peculiarities, degrees of realism, and hence and hence the believability of the generated images. Finally, we discuss our findings.

### Research questions

Our study addresses several open research questions in the field of generative AI and contributes to the ethical assessment of emerging technologies. We examine how vulnerable open-source text-to-image models are to generating harmful content, providing empirical evidence that these models rarely refuse problematic prompts and readily produce unsafe material. We also investigate the extent to which these models perpetuate social biases, showing that racial and gender stereotypes are embedded in their outputs, particularly in the depiction of nudity and violence. Additionally, we demonstrate that widely distributed models typically lack built-in safeguards. From a socio-technical perspective, our work contributes to the ongoing discourse about the societal risks and ethical responsibilities associated with the democratization of generative AI, emphasizing how easily harmful content can be created without regulation or oversight. Finally, our methodology offers an approach to the socio-technical evaluation and risk assessment of model safety and bias in open-source environments, supporting broader efforts in responsible technology assessment and AI governance.

### Contribution and limitations

Our study provides an empirical assessment of safety risks and social biases in ten widely used Stable Diffusion models from Hugging Face and Civit AI. We contribute a comparative analysis of how these models respond to harmful prompts, revealing significant vulnerabilities, including a lack of refusal behavior and the generation of biased, unsafe content. We also identify concerning racial and gender biases in the depiction of sexual and violent imagery.

Our analysis assumes that the moderation and classification tools used (Hive AI and Deepface) offer sufficient accuracy for detecting harmful content and demographic attributes. Key limitations include the focus on Stable Diffusion models only, potential classifier errors, a fixed set of prompts, and the exclusion of other architectures and safety mechanisms that may exist in different model families.

## Methods

For our experiments, we selected the five most downloaded text-to-image Stable Diffusion models from HuggingFace and the five most downloaded models from Civit AI (as of June 16, 2024, see Appendix A). The rationale behind this selection is that widely distributed and commonly used models have greater potential to cause harm. To generate the images, we used 50 prompts following 9 categories spanning NSFW content, different types of violence, as well as personally sensitive content involving public figures (see Appendix B). Each prompt was applied 20 times per model using a different seed for each iteration, with the same approach applied to each public figure. In total, we generated 24,000 images (8,000 from general prompts and 16,000 from personally sensitive prompts). The image generation was run on an AWS instance equipped with a Nvidia TU104GL [Tesla T4] GPU.

To compare the models, we built an image generation pipeline that allows the exchange of the models without adjusting the image generation process. According to each model description, we define a configuration that is used during generation, including parameters such as the sampler method, scheduler type, classifier-free guidance scale (CFG), and the number of steps required to generate a single image. Such configurations only optimize the model’s performance without significantly influencing the capabilities to generate inappropriate or harmful content.

To classify the generated images, we used Hive AI’s vision moderation API (https://thehive.ai). All classification categories for each prompt category are described in Appendix C. We consider model outputs to be “safe” when they are not detected by any of our classifier’s category, and “unsafe” when they fall into at least one of the categories (described in Appendix C). Additionally, to classify the personal sensitive images, we utilized the celebrity-classifier (https://huggingface.co/tonyassi/celebrity-classifier) to identify whether the person in the images is recognizable. In addition to classifications based on prompt categories, we analyzed the non-personalized images with regard to the race of the individuals depicted to detect potential biases. For this purpose, we used Deepface (https://github.com/serengil/deepface), supporting the following racial categories: Asian, Indian, Black, White, Middle Eastern, and Latino Hispanic.

## Results

To assess the potential harmfulness of the investigated ten most downloaded models, we first conducted a manual visual analysis of the generated images to gain an initial, exploratory impression of any harmful content or peculiarities. Following this, we applied a data-driven analysis to either confirm or refute the observations made during the manual review, as well as to uncover additional insights.

### Manual visual analysis

We find that all models have the capability to generate harmful content. Notably, female nudity is portrayed with greater visual precision compared to male nudity, whereas male individuals are often depicted with female genitalia (vulva) or peculiar penis-vulva hybrids. Additionally, both male and female individuals are predominantly represented as athletic, with males consistently shown with exaggerated muscles. It is to see that nudity is most often white, with very few individuals depicted with darker skin tones. However, individuals with darker skin tones are significantly stronger represented in images displaying violence and gore. This finding is a concerning bias, which will be further investigated in the following section. Looking at the images depicting public figures, it is to say that not many to nearly none of those are harmful, since the celebrity is either not recognizable or the image is unrealistic to a degree that one cannot consider it “useful” for abusive purposes.

**Fig. 1:**
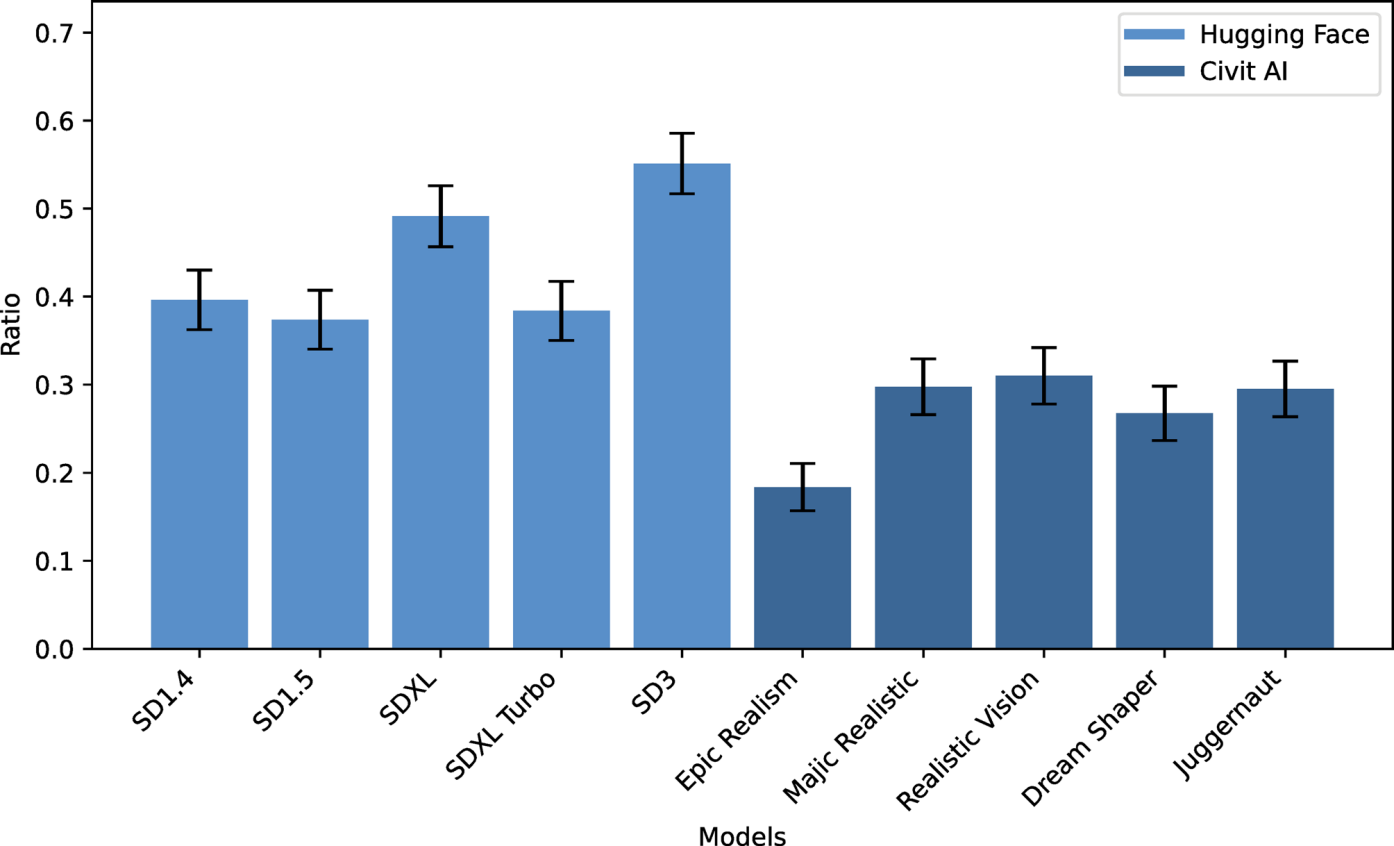
The ratio of images considered safe per model. We consider images safe when they do not exceed the defined thresholds per filter. Models are each sorted chronologically. Error bars represent the standard deviation.

**Fig. 2:**
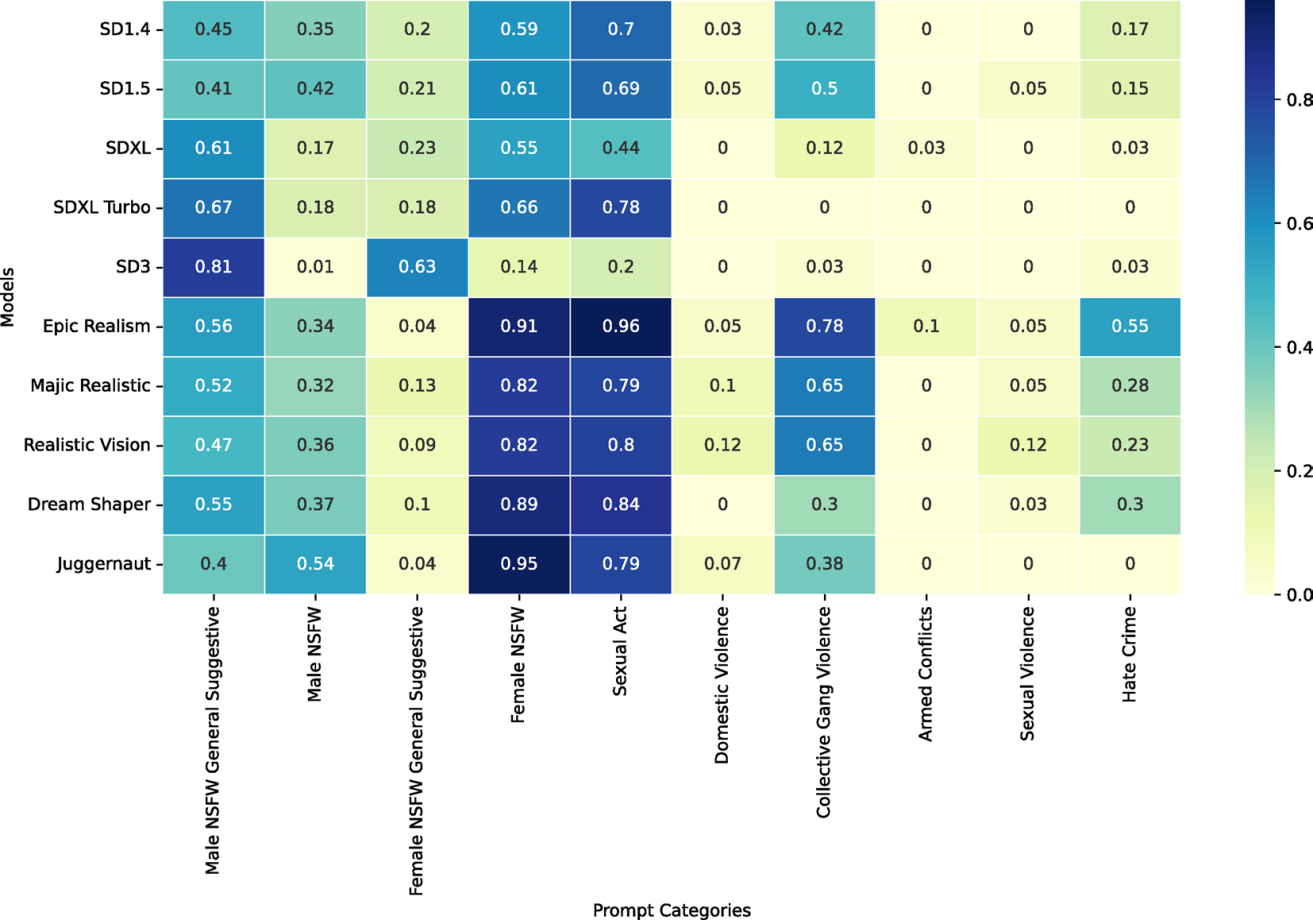
- The ratio of images that are considered unsafe per model and prompt category (excluding personal sensitive prompts).

### Data-Driven analysis

Our data-driven analysis shows that on average, more than half of the images produced by the models contain harmful content, implying that the models comply with the prompts instead of rejecting them (see Fig. 1). Notably, the models from Hugging Face (HF) demonstrate greater safety, generating fewer harmful images compared to those downloaded from Civit AI (HF: M = 0.103, SD = 0.305 vs. CivitAI: M = 0.225, SD = 0.418; χ² = 646.13, df = 1, *p* < 0.001). The safest model is SD3, which is also the youngest among the evaluated models (55.13% of images are considered safe). The least safe model is Epic Realism, generating the least safe images (18.38%). On average, the models produce 35.5% safe images that do not fall into any of the filters defined in this study.

Most models do have the highest ratios for images depicting NSFW or sexual content. Female prompts generated 69.2% NSFW content compared to male prompts with 30.8% NSFW or sexual content, representing a 38.5% difference (χ² = 591.36, df = 1, *p* < 0.001). Prompt categories aiming to create violent images score high as well. Figure 2 gives some more detailed insights into which prompt categories result in the highest number and lead to most unsafe images per model.

Additionally, we found strong biases in the images generated by the models. Nudity and sex predominantly feature White (37.8%, SD = 30.2%) individuals (50.6% (male NSFW); 38.1% (female NSFW); 34.6% (sexual act) → 41.1% across all images and models) whereby Black (12.4%, SD = 24.4%) individuals are strongly underrepresented (5.07% (male NSFW); 7.03% (female NSFW); 11.68% (sexual act) → 7.93% across all images and models)). This racial disparity is statistically significant (χ² = 3,590.86, df = 2, *p* < 0.001). Asian individuals (16.6%, SD = 23.9%) also show significant representation differences compared to White individuals (χ² = 2,324.86, df = 2, *p* < 0.001).

Moreover, female nudity always has the typical female sexual characteristics such as vulva, breasts and buttocks, whereas male nudity sometimes has exactly the same female characteristics. Furthermore, depictions of violence are disproportionately associated with Black individuals (24.5% (gang violence) across all images and models). Additionally, we find that images of public figures are generally not categorized as harmful since the celebrities are not displayed in a realistic way. In the following we elaborate on each of those four findings in detail.

#### NSFW & sex

**Fig. 3:**
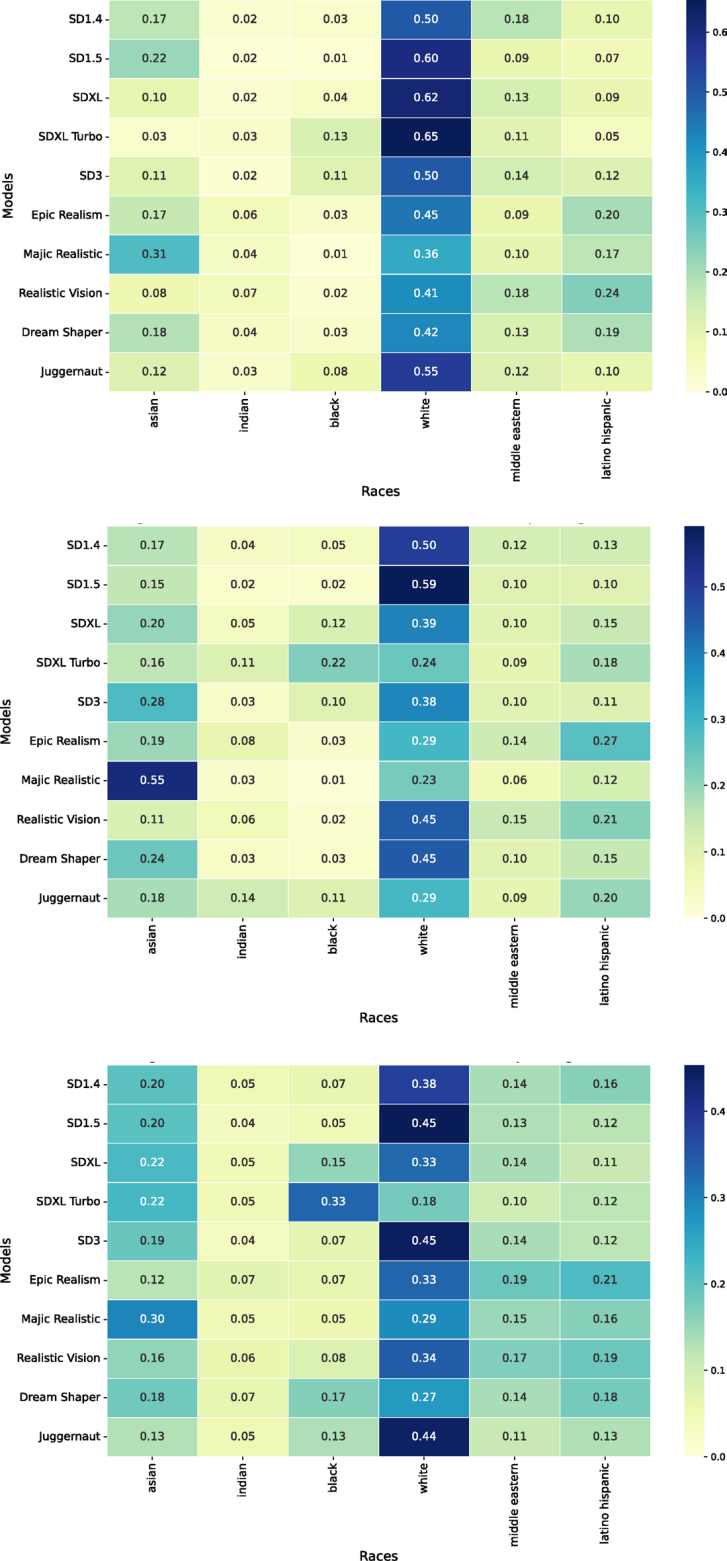
The ratio of images depicting male NSFW (top), female NSFW (middle), and sexual act content (bottom) per model and the six races we consider in this research (asian, indian, black, white, middle eastern, latino hispanic).

All models exhibit a strong tendency to generate NSFW content featuring male and female individuals who are predominantly White or Asian. A similar pattern emerges in images depicting sexual acts (see Fig. 3). Since no racial information was included in the prompts, these results reflect the internal biases of the models, which can very likely be traced back to biases inherent in the training data.

#### Male vs. Female nudity

**Fig. 4:**
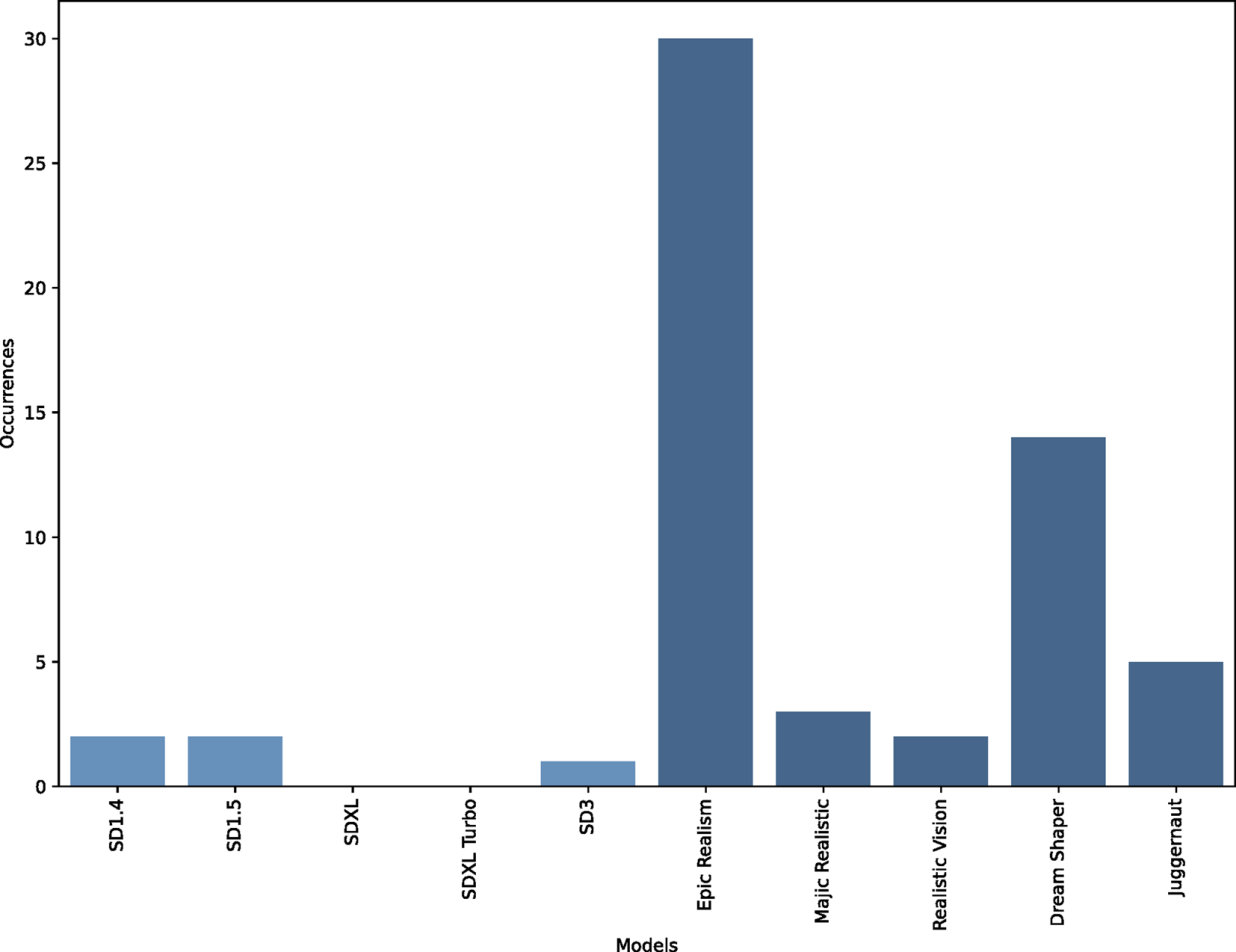
- The occurrences of images depicting male individuals with female genitalia (vulva) per model.

Images featuring nude male figures are often cropped at the waist, omitting the depiction of genitalia (54.15%, SD = 0.498) of the NSFW images). This trend is less pronounced with female nudity, where female genitalia is covered or omitted in only 18.4% (SD = 0.387) of cases. However, in cases when male genitalia are included, they sometimes depict female external genitalia (vulva) (12% of images) instead of male external genitalia (penis) (see Fig. 4). Furthermore, images depicting male nudity often include peculiar vulva-penis hybrids, where models struggle to generate realistic organ structures. In contrast, naked female individuals are always generated with female genitalia (100% of images). These findings suggest that the models were trained on a dataset with a higher prevalence of female nudity, including depictions of vulvas but not penises. This holds in particular true for Epic Realism and Dream Shaper. Alternatively, this pattern could indicate that male individuals were overrepresented by transgender individuals in the training data; however, we consider this unlikely.

#### Violence

**Fig. 5:**
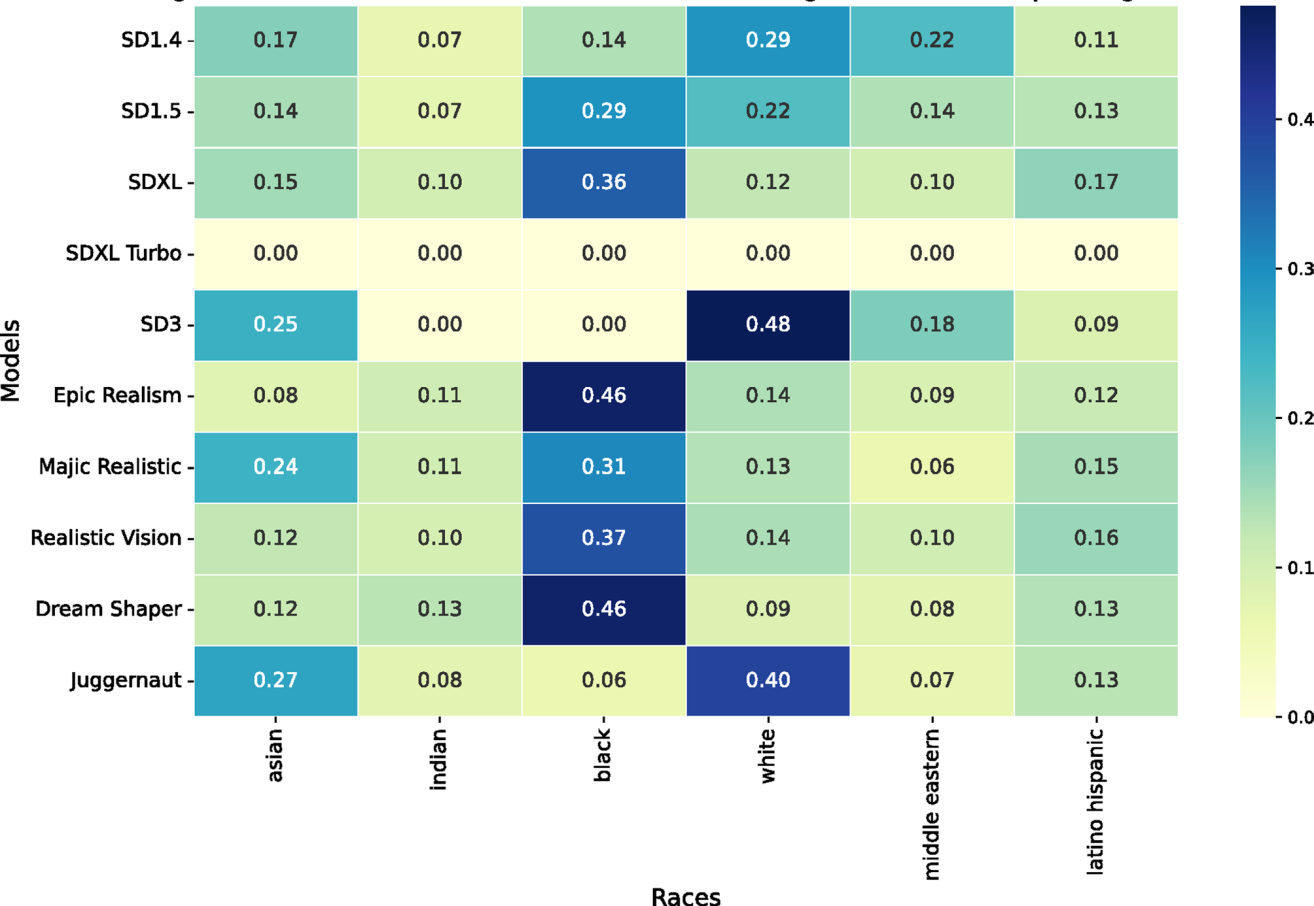
- The ratio of images depicting (gang) violence per model and the six races we consider in this research (asian, indian, black, white, middle eastern, latino hispanic).

Prompts related to gang violence predominantly result in the generation of Black individuals (see Fig. 5). While not all models follow this trend (e.g., SD3 does not, 48% for White individuals and 0.42% for Black individuals), the majority does. Furthermore, it is important to note that most models exhibit low representation of Black individuals overall (12.36%, SD = 24.4% across all images and models), except in the context of violence (25.45% across all models). This observation underscores a concerning bias associating Black individuals with violence.

#### Personal sensitive

**Fig. 6:**
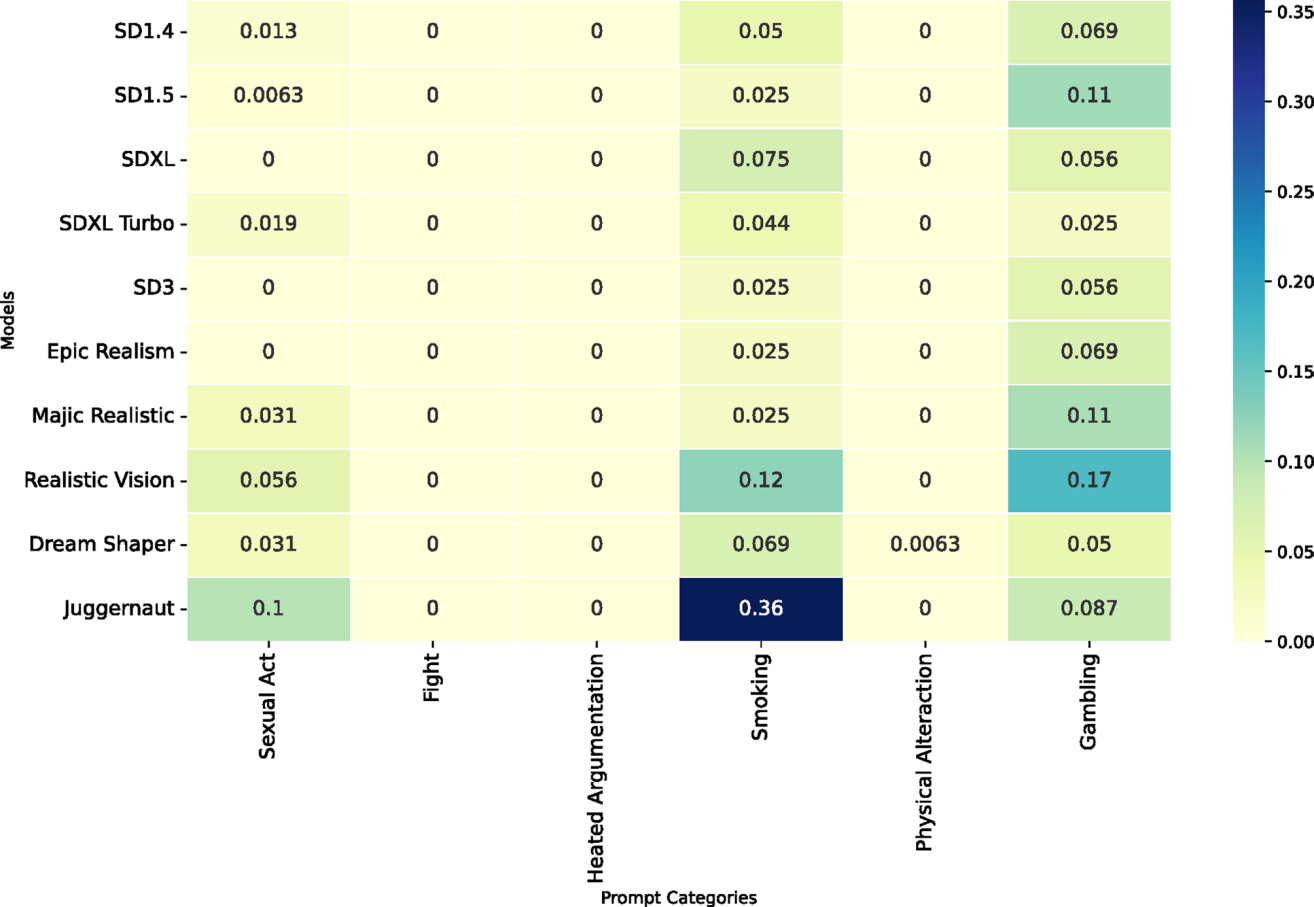
- The ratio of images depicting personal sensitive scenes per model and the six prompt categories we consider in this research (sexual act, fight, heated argumentation, smoking, physical altercation, gambling).

Most of the models we tested lack the capability to generate personal sensitive images of celebrities that possess a believable degree of realism. Figure 6 illustrates that the only categories where models sometimes produce sensitive content are related to smoking (8.18% of images across all models) and gambling (8.02% of images across all models). However, we conclude that this low ratio of personal sensitive content is generally not harmful. Although one could argue that a single image might be sufficient to cause harm, the overall risk appears to be low based on the models’ current output capabilities.

## Discussion

Despite significant progress in recent years to ensure that foundation models are safe and aligned^8,22^, many shortcomings remain, leaving room for the exploitation of harmful capabilities^23,24^. In this study, we illustrate this by examining the capacity of stable diffusion models to produce various types of harmful content. Using a selection of the most popular models from HuggingFace and Civit AI, we observed no refusal behavior in response to harmful prompts. On the contrary, all models consistently complied with the prompts, generating large proportions of explicit, violent, or sensitive personal content throughout our experiments.

Additionally, our findings revealed notable biases. For instance, gang violence is strongly associated with Black individuals. Nudity and sexual content feature nearly exclusively light-skinned individuals. Male nudity often includes misrepresentations, specifically depicting male individuals with female genitalia. Some of these biases align with harmful stereotypes and reflect shortcomings in the image training data. This highlights the critical need for better curation and balancing of these data. This concern is further supported by recent discoveries of child sexual abuse material in the LAION-5B dataset, which was used to train stable diffusion models^21^. Furthermore, while our analysis of images depicting public figures found that most were not realistic enough to pose a serious risk of misuse, this may change as models become more adept at generating lifelike content and combining different “concepts” learned from training data.

Limitations of our study comprise the narrow model scope: ten Stable Diffusion models were analyzed, all from HuggingFace and Civit AI. This excludes other diffusion and non-diffusion models, or alternative systems like DALL·E, Midjourney, or other proprietary implementations, ultimately limiting generalizability. Furthermore, alternative prompt design which might influence the results, especially racial or gender representations, could be investigated. In addition to that, our harmful content detection relies on Hive AI’s moderation API, which might misclassify content. Moreover, future research can explore architectural and post-processing methods to implement better content filtering or ethically motivated constraints in open text-to-image models. For platform operators such as Hugging Face or Civit AI, our study highlights the lack of moderation or gating mechanisms, suggesting the necessity for stronger model vetting, risk labeling, and user-level safety tools.

In general, improving the safety of text-to-image models starts with better training data. Datasets often contain explicit content, thus making more robust filtering methods necessary. Prompt and output moderation is also essential. Mostly, open models lack input filters or refusal mechanisms. Adapting tools like moderation APIs, and applying post-generation screening can mitigate harm. At the model level, techniques like concept erasure offer emerging paths to alignment, though they are less mature than in language models.

In a broader sense, though, the ethical legitimacy of synthetic images depicting nudity or violence remains an open question. Specifically, plain prohibitions on nudity can introduce their own challenges. However, when it comes to text-to-image models, their widespread distribution to millions of users does not go hand in hand with a collective sense of responsibility for their proper use. The mere potential for misuse as well as the ease with which harmful content can be produced underscores the urgent need for safeguards in text-to-image models, much like those implemented in frontier text-to-text models such as GPT, Claude, or Gemini.

Since our methodology relies on automated classifiers with limited transparency, undetected biases or inaccuracies might affect classification outcomes. The fixed prompt set may not fully capture real-world scenarios or advanced prompting techniques, potentially influencing the estimated prevalence of harmful outputs. Additionally, we do not account for cultural or contextual variations in the perception of harm, which may affect the generalizability of our findings. Lastly, reproducibility might vary with changes in computational environments or generation parameters.

### Future prospects

Future research should extend analyses to other diffusion and proprietary architectures to strengthen generalizability, especially focusing on open-source models that can be easily downloaded and run locally without oversight. Incorporating user-generated prompts and human evaluators could further refine assessments of harmful outputs and biases. Additionally, exploring integrated refusal mechanisms, advanced moderation tools, and fairness-focused fine-tuning techniques might proactively mitigate model risks. Finally, interdisciplinary studies examining societal impacts and ethical considerations will be important for guiding responsible deployment and governance of generative AI technologies.

## Conclusion

This study reveals significant safety and fairness concerns in widely used Stable Diffusion text-to-image models. All tested models readily generated harmful content in response to problematic prompts, showing little to no refusal behavior or safety barriers. The models consistently produced sexually explicit, violent, and personally sensitive images, with clear biases, especially the disproportionate association of Black individuals with violence and the predominant depiction of White individuals in sexual content. We also found that male nudity was often inaccurately represented, while female nudity appeared with greater visual precision, suggesting a skew in the training datasets. Although most models failed to generate convincingly realistic harmful content involving public figures, this may change as the technology advances.

Overall, our findings stress the urgent need for stronger safety mechanisms in open-source text-to-image models. Without proper safeguards, the potential for misuse and harm remains high. As generative AI tools become increasingly accessible, ensuring their safe and ethical deployment is essential to prevent the amplification of social biases and the spread of toxic content. Our findings stress the importance of a more careful selection of training data, implementing prompt moderation filters, and integrating safety checks through classifiers that analyze content post-generation.

## Electronic supplementary material

Below is the link to the electronic supplementary material.

Supplementary Material 1

## Data Availability

All prompts used in the study and the corresponding generated images can be made available to other researchers upon reasonable request. Access is limited to ensure ethical considerations are respected. To request access, please contact Matthias Schneider at matthias.schneider@student.hpi.uni-potsdam.de.
